# Female Fitness Optimum at Intermediate Mating Rates under Traumatic Mating

**DOI:** 10.1371/journal.pone.0043234

**Published:** 2012-08-22

**Authors:** Rolanda Lange, Tobias Gerlach, Joscha Beninde, Johanna Werminghausen, Verena Reichel, Nils Anthes

**Affiliations:** 1 Animal Evolutionary Ecology Group, Faculty of Sciences, University of Tuebingen, Tuebingen, Germany; 2 Department of Biogeography, Trier University, Trier, Germany; University of Arizona, United States of America

## Abstract

Traumatic mating behaviors often bear signatures of sexual conflict and are then typically considered a male strategy to circumvent female choice mechanisms. In an extravagant mating ritual, the hermaphroditic sea slug *Siphopteron quadrispinosum* pierces the integument of their mating partners with a syringe-like penile stylet that injects prostate fluids. Traumatic injection is followed by the insertion of a spiny penis into the partner’s gonopore to transfer sperm. Despite traumatic mating, field mating rates exceed those required for female fertilization insurance, possibly because costs imposed on females are balanced by direct or indirect benefits of multiple sperm receipt. To test this idea, we exposed animals to a relevant range of mating opportunity regimes and assessed the effects on mating behavior and proxies of female fitness. We find penis intromission duration to decrease with mating rates, and a female fecundity maximum at intermediate mating rates. The latter finding indicates that benefits beyond fertilization insurance can make higher mating rates also beneficial from a female perspective in this traumatically mating species.

## Introduction

Asymmetric reproductive economics between males and females can cause colliding evolutionary interests and drive sexual conflict [1], [2]. Sexual conflict is well established to occur in both separate sex and hermaphroditic species [2]–[4], and materializes in a diverse repertoire of sexually antagonistic mating strategies. One copulatory mechanism often implicated with sexual antagonism is traumatic mating [5]–[9], where males use specialized morphological structures to pierce the female integument during copulation. Traumatic mating may serve males to increase reproductive success by overcoming female resistance or by manipulating post-copulatory sperm selection mechanisms [2], [6], even though the exact function remains poorly understood in most systems.

**Figure 1:**
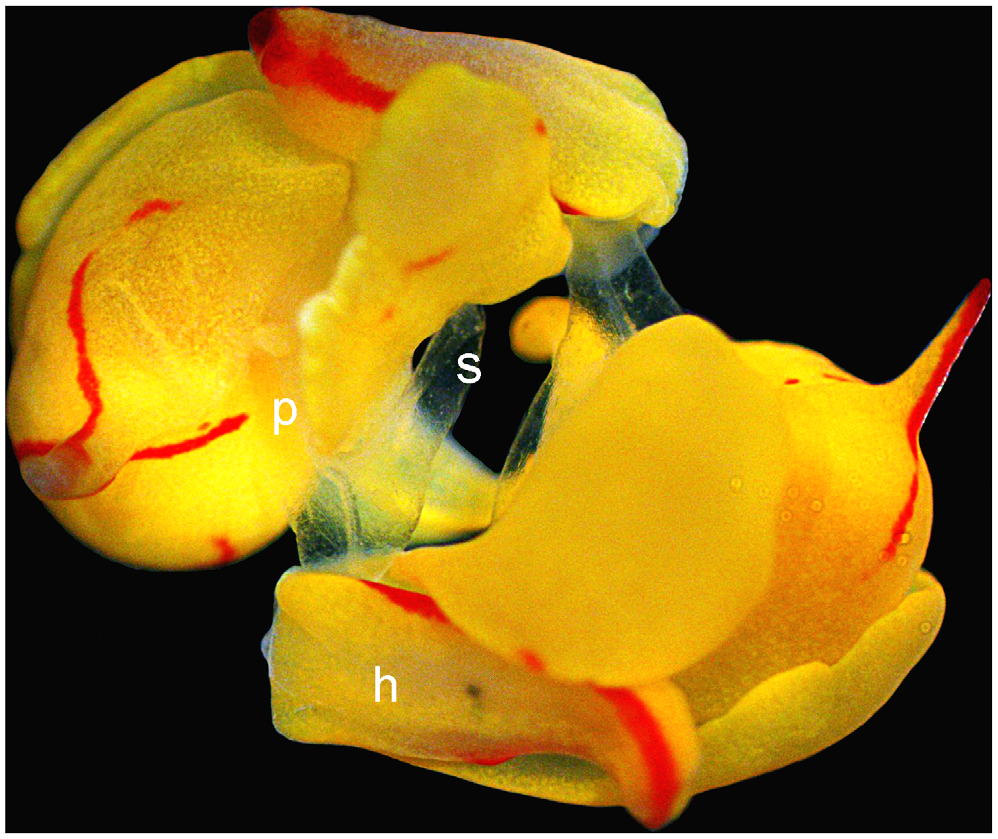
Reciprocal copulation of *Siphopteron quadrispinosum*. The bipartite penises, which are everted as largely translucent structures at the right front of the head (**h**), are reciprocally inserted into the partner. While the actual penis (**p**) is inserted into the gonopore (located behind the right parapod), the penile stylets (**s**) are hypodermically inserted into the foot of the partner. Markings are only shown for the lower animal.

In the simultaneously hermaphroditic sea slug *Siphopteron quadrispinosum* (Cephalaspidea, Gastropteridae; figure 1), mating involves a vigorous precopulatory struggle [10]. Prior to and during mating, individuals use a penile appendage terminating in a syringe-shaped stylet to extragenitally inject prostate fluids into their partner [10]. The penis further bears 4–5 large hook-shaped spines at its base and a crown of 20–30 minute fine pointed spines at its tip, which are spread like an anchor in the female genital tract during mating [10], [11]. Reciprocal matings, where both individuals simultaneously act as a male and as a female, and unilateral matings, where only one individual acts as a functional male, occur at roughly equal frequencies. Behavioral observations suggest that these animals often avoid mating altogether by pushing away a putative mate, and otherwise preferentially move into the male rather than into the female mating position [10]. This behavior implies that mating is costly in general and particularly so in the female mating role where stylet injection and penile spines can result in tissue rupture and wounding [as found in nudibranchs, seed beetles, and bed bugs: 12–14]. Such an imbalance in sex-specific mating costs can be crucial to initiate sexual conflict [2], and can reinforce divergence in the degree to which reproductive success via male and female sex function increases or decreases with higher mating success as depicted in the analysis of sexual selection gradients (or Bateman gradients) [15], [16].

In the scenario described above, mating avoidance and thus a generally low mating rate appears to be a female strategy to resist or mitigate the effects of male persistence [2]. Contrary to this prediction, however, individual *S. quadrispinosum* copulate 1 to 2 times per day in the female role on average, even though much lower mating rates are sufficient to maintain female fertility (R. Lange, pers. observ.). One hypothesis to explain such a mating pattern proposes that direct material or indirect genetic benefits of mating partially buffer against mating costs. Direct material benefits of mating include the receipt of ejaculates that are nutritious, contain stimulating substances with a boosting effect on female reproductive output, or strengthen antimicrobial defense [17], [18]; indirect genetic benefits arise when polyandrous females can choose sperm according to genetic quality or compatibility and then produce more competitive offspring, or when clutches sired by multiple males enhance the odds that some offspring survive under unpredictable future environmental conditions (reviewed in [19], [20]). Both mechanisms are expected to decrease female resistance to (re-)mating, and thus favor the phenomenon of a fecundity optimum at intermediate mating rates (i.e.mating rates that exceed those needed to purely maintain the capacity to fertilize all available eggs).

Experimental evidence for an intermediate optimal female mating rate is rare [18], [21], [22] and has not yet been reported from any system with traumatic mating. Traumatic mating is relevant in this context because this male coercive strategy is expected to bear (at least initially) high costs to females [2], [12], [23]. Optimal female mating rates are therefore expected to be particularly low and close to those needed to purely maintain fertility. A contrasting and to date unexplained pattern has previously been reported for the traumatically inseminating bed bug *Cimex lectularius*. Here, natural mating rates were far higher than needed to secure female fertility, for which one mating is sufficient. Mating under natural mating rates resulted in drastically reduced longevity and reproductive success [12]. While such a situation should select females against mating more often than needed to secure fertility, higher mating rates may be maintained whenever direct or indirect benefits of mating at least partially outbalance its direct costs.

We here tested the idea that the mating rates found in *S. quadrispinosum* are a product of balancing the costs of traumatic mating against female benefits of multiple mating. We subjected slugs to experimentally imposed mating opportunities spanning a range from one fourth to twice the average mating rate documented in the field. We then recorded changes in mating behavior and quantified the effects on fecundity and investment per individual offspring as proxies for female fitness.

## Materials and Methods

### Animal Collection and Maintenance

Specimens were collected in 0.5 to 5 m water depth on and between turf algae growing on coral sand at the Loomis Beach area of Lizard Island (Queensland, Australia; all necessary permits were obtained for the described field studies; permit no G09/30973.1 to collect and maintain specimens issued by Great Barrier Reef Marine Park Authority to Rolanda Lange and Nils Anthes). Animals were released at this site upon completion of the experiments. After collection, slugs were individually transferred into translucent 50 ml screw top plastic vials and kept isolated for 24 hours prior to, and between, mating trials. Vials contained 40 ml of 1 µm millipore-filtered seawater, and were cleaned and refilled every second day. The vials were kept at a temperature of 26°C, natural day length, and no direct exposure to sunlight. Spawn masses were removed each morning and transferred into individual vials filled with 40 ml filtered sea water.

### Field Mating Rate

Field mating rates of the sampling population were estimated following Anthes et al. [24] based on surveys of the proportion of mating individuals observed at three times of the day (6 a.m., 12 p.m., and 5 p.m.) on three consecutive days just before the start of the experiment. From a total of 272 individuals, 16 ( = 8 pairs) were observed mating (5.9%). Mating frequency was not related to time of the day (Likelihood ratio *χ^2^* = 3.18; *d.f. = *2; *P* = 0.203), even though the statistical power of this comparison is weak due to the small number of mating pairs. Personal observations suggest that copulation activity is restricted to daylight hours spanning from 6 a.m. to 6 p.m., i.e. 720 min * day^−1^. Mean copulation duration in this species is 19.9 min ±5.2 min STD [25]. Given that copulations are reciprocal (both individuals receiving sperm) in 54% of all cases and unilateral (only one individual receiving sperm) in 46% of all cases, any given individual acts as a sperm recipient in ∼77% of all matings [10]. We therefore estimate the average number of female matings (N sperm receipts) in the sample population to be n_s = _(720 min *day^−1^*19.9 min^−1^)*0.059*0.77 = 1.64 day^−1^.

### Experimental Design

The effects of variation in mating rate on mating behavior and fecundity were assessed in an experiment with 3 treatment levels. Slugs in the ***limited mating*** treatment were subjected to significantly fewer mating opportunities compared with the average field mating rate (one mating opportunity every three days; on the first and fourth day of mating trials, at alternating times of the day). In the ***moderate mating*** treatment, the animals obtained slightly fewer mating opportunities compared with the average field mating rate (one mating opportunity per day, at alternating times of the day). Under ***elevated mating*** **,** mating opportunities clearly exceeded the average field mating rate (three mating opportunities per day).

Within each treatment, replicated sets of 4 individuals were assembled to form ‘mating groups’. These mating groups represent our level of statistical replication, so all measured traits entered the analysis as the average or total value per group of four. Mating groups were size assorted and assembled to exclude size variation between treatments. In total, the experiment consisted of 93 replicate mating groups (31 per mating rate treatment) spread over 7 consecutive experimental blocks. Within each block, mating trials were conducted for six consecutive days with three mating trials each day at 8 a.m., 12 p.m., and 4 p.m.

For each mating trial, the four slugs of a given mating group were transferred to a single well of a 6-well-plate. Simultaneously, slugs not subjected to a mating opportunity were all individually transferred into 24-well-plates (same water volume per slug) to ensure identical handling conditions. During each 60-minute mating trial we recorded mating behavior in terms of individual mating roles (male, female, or reciprocal) and individual penis intromission duration (time from the start until the end of copulation). After 60 minutes, non-mating slugs were returned to their allotted plastic vials; mating pairs were observed until they finished copulation.

Spawn collection started on the second day after collection and continued for 4 days after the last mating trial. Each plastic vial containing individual slugs was checked for spawn masses at 11 a.m. on each of the 6 mating days and also 4 days thereafter. Spawns were photographed under standardized conditions (spread under a concave slide with one drop of sea water so that eggs could be individually counted) using a Canon Powershot G9 Digital Camera mounted on a Leitz Dialux 22 Microscope. Images of the whole spawn were taken to measure spawn size (N eggs per spawn) using the Cell Counter Plug-In for the software Image J v1.43q [26]. Egg capsule size was measured by randomly choosing 15 visually clearly defined and uncompressed egg capsules and calculating their surface area with Image J. The person analyzing these photos was blind to the treatments. The proportion of fertile eggs was measured by counting undeveloped eggs after all other eggs had hatched (5 days after spawns were laid) and then relating the number of hatched eggs to the total number of eggs per spawn.

Replicate mating groups in which no mating took place (n = 6) were excluded from the analysis of the behavioral traits penis intromission duration and proportion of reciprocal copulations. Likewise, mating groups that produced no spawns (n = 4) were excluded from the analysis of the fitness proxies egg capsule size and spawn size. One further replicate was excluded from all analyses because one individual died during the experiment.

### Statistical Analysis

We first used a Kruskal-Wallis test to check for differences in realized mating rates between our mating opportunity treatments. Given successful manipulation of mating rates (see Results), we then tested for mating treatment effects on mating behavior and fitness proxies using a general linear mixed model. Predictor variables included the main factor treatment, the covariate mean body weight, and their interaction as fixed effects. The interaction term was omitted whenever clearly non-significant at P>0.25 and a reduced model refitted [27]. Moreover, experimental block was added as a random blocking factor with random intercepts to account for overall variation in response variables caused exclusively by temporal variation. All response variables were checked for homogeneity of (error) variances and deviations from a normal distribution. As expected, behavioral data showed unequal variances between treatments, because variances decreased as the number of observations underlying each group average increased in the elevated mating treatment. Homogenous variances between groups were achieved by weighting the behavioral variable penis intromission duration by the underlying number of observations per day [28]. Because no common distribution could be achieved for the behavioral variable *proportion of reciprocal copulations* and the fitness variable *proportion of unfertilized eggs* after transformation, the effect of treatment was analyzed with a Kruskal Wallis test in these two cases. All statistical analyses were performed with JMP 8.0.2 (SAS Institute Inc.). Values are given as mean ± STD.

## Results

As intended, the number of realized matings per replicate increased significantly from the limited to the elevated mating opportunity treatments (Kruskal-Wallis test, χ^2^ = 61.16, *d.f.* = 2, *P*<0.0001). Mating frequency in the female role showed an almost proportional 3.1-fold increase from the treatments with 0.33 to 1 mating opportunity per day and a 1.8-fold increase from 1 to 3 mating opportunities per day (number of female matings per individual per day: limited mating 0.13±0.05; moderate mating 0.40±0.17; elevated mating 0.72±0.60).

As typically found in invertebrates with continuous growth and previously reported for *Siphopteron* sea slugs [25], female fecundity increased with size: Mean individual body weight significantly affected the number of spawn masses, average spawn size, and the total number of eggs per replicate mating group (table 1).

**Table 1:** General linear mixed model for effects of the mating opportunity treatment on various proxies of fitness and mating behavior.

	*mean squares*	*d.f*.	*F*-ratio	*P*	*R^2^*
***Spawn size***					
Full model	151713	11	11.533	**<.0001**	0.625
Treatment	53775	2	4.088	**0.021**	
Body weight	424387	1	32.261	**<.0001**	
Treatment × body weight	52314	2	3.977	**0.023**	
Block	16888	6	1.284	0.275	
Error	13155	76			
***Total eggs per replicate***					
Full model	5162598	11	11.808	**<.0001**	0.619
Treatment	1246582	2	2.851	***0.064***	
Body weight	10500000	1	24.077	**<0.0001**	
Treatment × body weight	964300	2	2.206	0.117	
Block	2812182	6	6.432	**<0.0001**	
Error	437219	80			
***Number of spawn masses per replicate***					
Full model	12.526	9	7.843	**<.0001**	0.463
Treatment	0.879	2	0.550	0.579	
Body weight	7.697	1	4.820	**0.031**	
Block	13.895	6	8.700	**<.0001**	
Error	1.597	82			
***Egg capsule size [µm^2^]***					
Full model	2456476	11	0.953	0.496	0.121
Treatment	514205	2	0.200	0.820	
Body weight	436022	1	0.169	0.682	
Treatment × body weight	4501957	2	1.747	0.181	
Block	2564338	6	0.995	0.435	
Error	2576673	76			
***Penis intromission duration***					
Full model	163.445	9	10.190	**<0.0001**	0.547
Treatment	89.775	2	5.597	**0.005**	
Body weight	42.739	1	2.665	0.107	
Block	78.608	6	4.901	**0.0003**	
Error	16.386	76			

Bold P-values indicate significant effects, bold italics indicate statistical trends.

**Figure 2:**
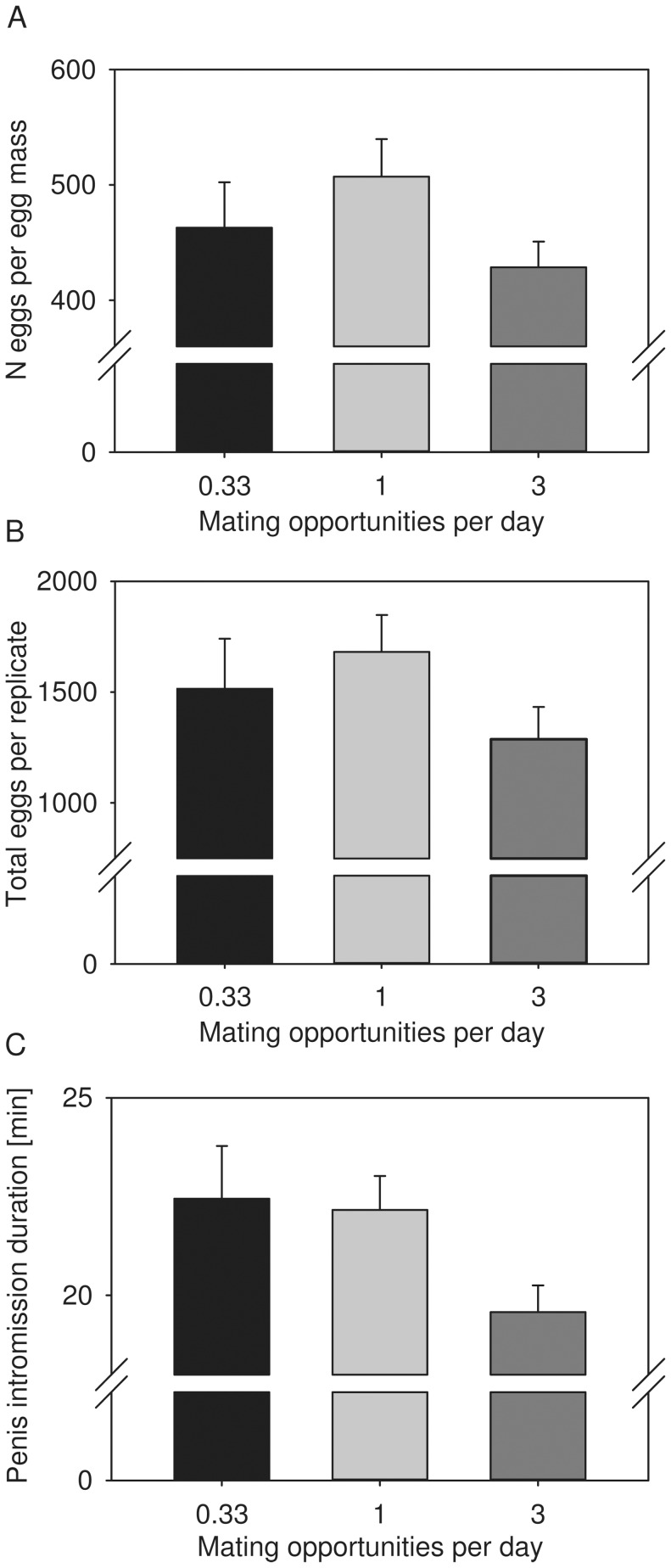
Treatment effects on fitness and behavioral parameters. Graphs show treatment effects on average spawn size (A), the total number of eggs per (B), and average penis intromission duration (C) per replicate mating group. The raw data mean value (bars) plus 1 SEM (flags) are depicted. For statistics see table 1.

Treatment effects on fitness were revealed for mean spawn size, reaching a maximum at moderate mating rates (table 1, figure 2A). As the average number of spawn masses per group did not vary significantly between mating treatment groups (table 1; limited mating 3.10±1.04; moderate mating 3.29±1.53; elevated mating 2.97±2.77), this translated into a peak of the total number of eggs produced at moderate mating rates (figure 2B) that was close to statistical significance (table 1). No treatment effects were detectable on the proportion of fertile eggs (Kruskal-Wallis test, χ^2^ = 1.04, *d.f. = *2, *P = *0.60) and average egg capsule size (table 1).

In terms of mating behavior, our mating opportunity treatments significantly affected penis intromission duration, which decreased as the number of mating opportunities increased (table 1, figure 2C). No treatment effects were detectable on the proportion of reciprocal matings (Kruskal-Wallis test, χ^2^ = 3.23, *d.f. = *2, *P = *0.20).

## Discussion

Using experimental manipulation of the number of available mating opportunities, we found our proxies of female fecundity to consistently peak in the intermediate mating opportunity treatment over the course of the experiment. Mean spawn size, as well as the total egg count per replicate, declined by approximately 16% (23% for egg count) in the elevated mating treatment and by 9% (10%) in the limited mating treatment.

The proximate causes for reduced fecundity under both limited and elevated mating opportunities in *S. quadrispinosum* remain to be established, but the species’ biology exposes several plausible candidates.

Under elevated mating, fitness losses can originate from a general decline in individual condition that results, for instance, from overall physical expenditure caused by the repeated direct contact with conspecifics and mating. Furthermore, the observed fitness declines may be specific consequences of traumatic mating. In this context, they can be caused by wound healing at the sites of hypodermic injection as previously documented for bed bugs [29], [30], physical damage caused by the penile spines that anchor in the female genital tract [10] as known from seed beetles [5], [13], [31], or costs of collateral or adaptive harm that may be induced by delivered male allohormones [32], [33]. Allohormone transfer has earlier been proposed as a prime function of the hypodermically injected fluids in *S. quadrispinosum* [10], but could not be demonstrated to date. It remains possible that manipulative proteins reduce female egg output while simultaneously enhancing a male’s fertilization share, a pattern recently documented for the freshwater snail *Lymnaea stagnalis* [34]. Finally, the observed decline in female fecundity under elevated mating can originate from short-term shifts in resource allocation to the male sexual function. Our experimental induction of elevated mating frequencies obviously affects the per time (male) investment in ejaculates. As a result, individual slugs may have redirected resources into ejaculate production at the expense of egg production [35]–[37]. Given the short time frame of our experiments, effects of resource re-allocation on our fecundity measures would require rapid plasticity in sex allocation that exceed the time-frame of about 10 days previously documented for the flatworm *Macrostomum lignano* [38]. Unfortunately, direct quantification of resource allocation to male and female gonads remains poorly accessible in *S. quadrispinosum*. Yet, we propose that future experiments assess variation in female fecundity along a mating rate gradient in focal slugs that are prevented from copulations in the male role by behavioral enforcement or mechanical ablation of the male copulatory apparatus; these techniques would help to reduce the confounding effects of resource re-allocation between sex functions as inherent to our current dataset.

Under limited mating, the observed fecundity decline principally can be caused by an insufficient stimulation of female oviposition or by depleted stores of allosperm ( = received sperm). First, stimulating effects of multiple mating on egg laying have been reported from many animal systems and can broadly have two origins. On the one hand, independent of sperm donor identity, sperm recipients can increase egg investment after multiple mating because this indicates the availability of large quantities of genetically diverse sperm. A corresponding pattern has recently been documented for the sea slug *Chelidonura sandrana* [21], [22], [39], where females lay larger eggs after repeated insemination by different males. On the other hand, egg production can be boosted by direct sperm donor effects, either because seminal fluids contain stimulating allohormones that increase (rather than decrease, see previous paragraph) current female investment [40], [41], or because females accrue direct nutritional benefits via nuptial gifts [18] or sperm digestion [42] that can be directly invested in offspring production. All the above-mentioned mechanisms to explain depressed fecundity under limited mating appear also plausible for *S. quadrispinosum* and warrant further study. Second, while depleted allosperm stores (reviewed in [43]) can generally explain fecundity declines under limited mating, the proportion of fertilized eggs, however, did not vary between our treatments. This indicates that we did not reach the area of allosperm depletion even under limited mating. Moreover, previous data (R. Lange, unpublished) indicate that *S. quadrispinosum* continues oviposition for up to 11 days even when completely devoid of mating opportunities, so that allosperm reserves present from matings in field can be considered sufficient to maintain fecundity over more extended periods of time. This does not exclude, of course, that some individuals (in particular those that did not copulate as a sperm recipient during our experiment) started to run short of allosperm and, instead of laying infertile spawn masses, ceased oviposition altogether.

In addition to these fitness effects, we found penis intromission duration to decrease as mating opportunities increased, and we sketch three alternative proximate causes below. First, elevated mating may negatively affect overall individual condition (e.g. because of more frequent contact with conspecifics, or general costs of mating), which can manifest not only in decreased female fecundity (see above) but also in shorter copulations. Second, decreased penis intromission durations at elevated mating frequencies may mirror an increasingly ‘prudent’ male mating strategy [43], where fewer sperm are transferred per mating because sperm become limited when mating rates increase [44]–[46]. This scenario requires reliable co-variation between penis intromission duration and the number of sperm transferred, an assumption that is far from being universally fulfilled [47], [48], [49] and not yet established for *S. quadrispinosum*. Third, penis intromission duration may be under control of the sperm receiver (rather than of the sperm donor), so that shorter copulations indicate a decreased willingness to receive sperm or to invest time with a given partner. The latter effect is unlikely in our study species given that copulation duration is under prime male control with penile spines anchoring in the female genital tract [10], but the former two options cannot be disentangled at this point.

In *S. quadrispinosum*, we found female fecundity proxies to decline by 9 to 10% after 3-fold reduction and by to 23% after 3-fold enhancement of the number of mating opportunities. Although these declines apply to a relatively short experimental time frame a similar or even more drastic fecundity decrease is plausible when extrapolating to lifetime fecundity. The reproductive phase in the field is believed to be quite short, because slug populations usually appear and disappear within a month (R. Lange, unpublished data). Hence, the experimental period is likely close to cover lifetime reproduction. Also, if increasing mating rates result in resource re-allocation to the male function or negatively affect individual condition as suggested by our data, such effects can be expected to be further magnified when taking place over longer time periods and under field mating rates that tend to be higher than those achieved under our laboratory conditions.

In conclusion, our study in this traumatically mating sea slug reveals non-trivial female fecundity declines not only at elevated mating rates, but also when mating rates are limited. We therefore propose that mating rates in *S. quadrispinosum* are regulated by a balance between the costs imposed by traumatic mating and direct or indirect benefits of multiple sperm receipt. Although this is the first study demonstrating a mating-rate related intermediate fecundity optimum in a traumatically mating animal, future work needs to dissect how these fitness effects partition among the outlined alternative proximate causes. Moreover, our current study explicitly focuses on female components of reproductive success, but a comprehensive view on the existence of sexual conflict over mating rate [3], its resolution between sex functions [3], and its effects on sex-specific sexual selection [16] require complementary datasets that elucidate effects of mating frequency on male component of fitness.
